# Upregulation of miR-200b Inhibits Hepatocellular Carcinoma Cell Proliferation and Migration by Targeting HMGB3 Protein

**DOI:** 10.1177/1533033818806475

**Published:** 2018-10-21

**Authors:** Long-kun Wang, Xi-Na Xie, Xu-Hong Song, Ting Su, Xiao-Lan Chang, Man Xu, Bin Liang, Dong-Yang Huang

**Affiliations:** 1 Department of Cell Biology and Genetics, Key Laboratory of Molecular Biology in High Cancer Incidence Coastal Chaoshan Area of Guangdong Higher Education Institutes, Shantou University Medical College, Shantou, China; 2 Institute of Translational Medicine, Shenzhen Second People Hospital, The First Affiliated Hospital of Shenzhen University, Health Science Center, Shenzhen, China

**Keywords:** HMGB3, miR-200b, human hepatocellular carcinoma, tumor progression, prognosis

## Abstract

HMGB3 belongs to the high-mobility group box subfamily and has been found to be overexpressed in gastric cancer. However, the expression and the role of HMGB3 in human hepatocellular carcinoma remain unknown. Here, we report that HMGB3, which is suppressed by miR-200b, contributes to cell proliferation and migration in human hepatocellular carcinoma. After analyzing The Cancer Genome Atlas data of 371 patients with hepatocellular carcinoma, we identified HMGB3 to be upregulated in human hepatocellular carcinoma tissue. Knockdown of HMGB3 in the hepatocellular carcinoma cell line suppressed cell proliferation and migration. TargetScan analysis showed miR-200b to be a possible regulator for HMGB3. Subsequent luciferase assays indicated that HMGB3 was a direct target of miR-200b. In addition, upregulation of miR-200b inhibited hepatocellular carcinoma cell growth and migration. HMGB3 overexpression or miR-200b downregulation was associated with poor prognosis. Our findings suggest HMGB3 may serve as an important oncoprotein whose expression is negatively regulated by miR-200b in hepatocellular carcinoma.

## Introduction

Hepatocellular carcinoma (HCC) is one of the most generally diagnosed malignant tumors worldwide.^1^ The vast majority of patients develop liver cancer due to cirrhosis and chronic inflammation caused by viral hepatitis B and C, aflatoxins, or ethanol.^2,3^ At present, surgical resection and transplantation are the only effective methods for treatment of HCC.^4^ Local tumor resection is the choice of treatment for separate HCC in noncirrhotic cases, although this condition is not universal in Western countries.^5^ However, the recurrence rate within 5 years in patients who have undergone tumor resection remains more than 70%, mainly because of intrahepatic dissemination of primary lesions (60%-70%).^6,7^ Therefore, it is necessary to develop new therapeutic strategies based on greater understanding of the molecular mechanisms of HCC proliferation and metastasis.

HMGB3, also named HMG2a, is a member of the X-linked high-mobility group subfamily of HMG proteins. It has 80% identity with HMGB1 and HMGB2.^8^ The high-mobility group box (HMG-box) subfamily is a group of chromosomal proteins that are involved in DNA replication, recombination, transcription, and repair.^9-11^ HMGB3 plays a critical role in leukemia stem cell renewal by activation of MLL/Hox/Meis. In addition, HMGB3 overexpression positively correlates with poor leukemia prognosis due to unconventional self-renewing progenitor-like cancer stem cells.^12^ Enhanced expression of HMGB3 also occurs in gastric adenocarcinoma, and HMGB3 RNA interference inhibits gastric cancer cell growth.^13^ In urinary bladder cancer, upregulation of HMGB3 is also linked with poor prognosis, and HMGB3 enhances cell proliferation and migration in urinary bladder cell lines and proliferation and invasion in breast cancer cell lines.^14,15^ Recent studies show that HMGB3 plays a carcinogenic role in colorectal cancer.^16^ Therefore, HMGB3 participates in the progression of leukemia, gastric adenocarcinoma, urinary bladder cancer, breast cancer, and colorectal cancer. To the best of our knowledge, involvement of HMGB3 in HCC has not been reported.

MicroRNAs (miRNAs) are a class of evolutionarily conserved noncoding RNAs that silence their target messenger RNAs (mRNAs) by binding sequence-specific sites on the 3′-untranslated region (UTR) of the target mRNA.^17,18^ MicroRNAs function as important posttranscriptional regulators of key genes in various biological processes, such as proliferation, apoptosis, differentiation, and carcinogenesis.^19^ Increasing evidence suggests that miR-200b acts as a tumor-suppressive miRNA by targeting certain oncogenic genes, and abnormal expression of miR-200b has been found in various tumor types, such as HCC, nasopharyngeal carcinoma, prostate cancer, and lung adenocarcinoma.^20-23^ Using bioinformatic tools, we predicted that HMGB3 was a potential target for miR-200b. In this study, we show that HMGB3 enhances proliferation and migration in HCC and is negatively regulated by miR-200b.

## Materials and Methods

### Cell Culture

Human HCC cell lines HepG_2_ and 7402 cells and the normal human hepatocyte cell line (HH) liver cell line were obtained from the Shanghai Cell Bank of the Chinese Academy of Science. All cell lines were grown in medium supplemented with streptomycin and penicillin (Sangon Biotech, Shanghai, China) and 10% fetal bovine serum (FBS; Gibco-BRL, Grand Islandn, NY) at 37°C with 5% CO_2_ in a humidified atmosphere.

### Transient Transfection

For transient transfection, HepG_2_ cells were inoculated at 3.0 × 10^5^ cells/well in 6-well plates and were transfected with miR-200b mimics or a negative control (NC; GenePharma, Shanghai, China) at a final concentration of 25 pmol/L using RNAiMAX (1946851; Invitrogen, Carlsbad, CA) according to the manufacturer’s instructions. Transfection efficiency was verified by quantitative real-time polymerase chain reaction (qRT-PCR). For the silencing experiments, HepG_2_ cells were transfected with small-interfering RNA (siRNA) targeting HMGB3 (siRNA-HMGB3) or their NC (siRNA-NC; GenePharma) using RNAiMAX. The sequence of the 21-nucleotide siRNA targeting human HMGB3 mRNA (5′-GACUAUAAGUCGAAAGGAATT-3′) was designed by GenePharma. Subsequent experiments were performed 48 hours after transfection.

### RNA Extraction and qRT-PCR

Total RNA, including miRNA, was extracted and purified from tissue samples and cell lines using a miRcute miRNA Isolation kit (DP501; Tiangen, Beijing, China). Reverse transcription of 2 ng RNA to complementary DNA (cDNA) was performed using a miRcute plus miRNA First-strand cDNA synthesis kit (KR211-02; Tiangen). TRIzol reagent (9109; TaKaRa, Dalian, China) was used to extract cell RNA, and reverse transcription was conducted with a PrimeScript RT Reagent Kit (RR047A; TaKaRa) according to the manufacturer’s instructions. The following primers were used: HMGB3 forward, 5′-GACCAGCTAAGGGAGGCAA-3′, and reverse, 5′-ACAGGAAGAATCCAGACGGT-3′; and β-actin forward, 5′-CTGGAACGGTGAAGGTGACA-3′, and reverse, 5′-AAGGGACTTCCTGTAACAATGCA-3′; miR-200b-3p primers (CD201-0281) and control U6 primers (CD201-0145) were provided by Tiangen Biotech Co, Ltd. HMGB3 was amplified as follows: denaturation at 95°C for 5 minutes, and then 40 cycles at 95°C for 10 seconds and 60°C for 30 seconds. MiR-200b was amplified in an ABI 7500 Real-Time PCR System (Applied Biosystems, Waltham, MA) as follows: denaturation at 95°C for 15 minutes, and then 40 cycles of 94°C for 20 seconds, 60°C for 34 seconds. The small nucliear ribonucleic acid (snRNA) U6/β-actin served as normalization groups and the relative expression levels were assessed using the 2^−ΔΔct^ method.^24^

### Plasmid Construction and Luciferase Reporter Assays

A 192-bp fragment from the HMGB3 3’UTR (position 741-942) containing the binding sequences of miR-200b was amplified by PCR from HepG_2_ cell genomic DNA. Also, a 105-bp fragment from the HMGB3 3’UTR (position 252-361), with the miR-200b target site deleted, was synthesized by PCR from HepG2 cell genomic DNA. The fragments were then cloned into the SacI and XhoI restriction sites downstream of the luciferase reporter gene in the pmirGLO vector (E1330; Promega, Madison, WI). Primer sequences for amplification of 2 HMGB3-3′-UTR segments were as follows: HMGB3 3′UTR (position 741-942) forward, 5′- CGAGCTCGAAGTTCAAGAACCTCCTGTA-3′, and reverse, 5′-CCCTCGAGGGGCCAGTCTCCAAATACAATG-3′; HMGB3 3’UTR (position 252-361) forward, 5′-CGAGCTCGACTCTCGTGTTCTCCTCA-3′, and reverse, 5′-CCCTCGAGGGCCATAGTTACCACCACCTTA-3. HepG_2_ cells were transiently cotransfected with miR-200b mimics, or the NC miRNA (miR-NC), and the target site reporter gene—HMGB3 3′-UTR vector—or target site deleted reporter gene HMGB3 3′-UTR vector. Luciferase activities were measured using a Dual-Luciferase Reporter Assay system (E1910; Promega) according to the manufacturer’s instructions 48 hours after transfection.

### MTT Assay

HepG_2_ cells were plated at a density of 5 × 10^3^ cells/well in 96-well plates in 100 μL of medium. After si-HMGB3 or miR-200b, mimics were transfected for 24, 48, 72, and 96 hours, 5 mg/mL MTT reagent (T0793; AMRESCO, Solon, OH) was added to each well and the plates were incubated at 37°C for a further 4 hours. Then, the MTT was extracted with 150 μL dimethyl sulfoxide (DMSO [Sigma-Aldrich, St. Louis, MO]) and the absorbance was recorded at 490 nm with a spectrophotometer. Each assay was performed using 5 replicates per sample in 3 independent experiments.

### Cell Growth Curve

The 3.0 × 10^5^ HepG_2_ cells were inoculated in 6-well plates in 2 mL of medium. After si-HMGB3 or miR-200b mimics were transfected for 24, 48, 72, and 96 hours, the cells were trypsinized and manually counted. For each plate, cell count was repeated 3 times to draw the cell growth curve.

### Colony Formation Assays

Colony formation assays were used to evaluate the clonogenicity of HCC cells following different treatments. Transfected cells (1000 cells per well) were planted in 6-well tissue culture plates 48 hours after transfection. After incubation for 10 days, the medium was discarded, and each well was washed twice with phosphate-buffered saline (PBS). The cells were fixed in 4% paraformaldehyde for 30 minutes and stained with Crystal violet for 15 minutes. After washing again, the plates were photographed under a microscope.

### Wound Healing Assay and Transwell Migration Assay

For the wound healing assay, transfected HepG_2_ cells in 6-well plates were cultured to confluence. Then, cells were treated with 10 mg/mL mitomycin C (CB47589; MERCK, Shanghai, China) for 4 hours to inhibit growth,^25^ followed by wounding with a sterile plastic pipette. Cells were washed twice with PBS to remove cellular debris, and then the monolayer was subsequently maintained in dulbecco's modified eagle medium (DMEM) supplemented with 1% FBS and cultured for 48 hours. The wound was photographed at time 0 and 48 hours. For the transwell migration assay, at 48 hours posttransfection, 1 × 10^5^ HepG_2_ cells were suspended in DMEM without FBS and inoculated in the upper chamber of a transwell (8 µm pore size, 6.5-mm diameter; Corning, Shanghai, China), with DMEM containing 10% FBS in the lower chamber. After incubation for 48 hours at 37°C, cells in the upper chamber were carefully removed with a cotton swab, and migrating cells were fixed in 4% paraformaldehyde, stained with Crystal violet, and 5 random fields counted at 100× magnification.

### Western Blotting

Total protein from cell lines was extracted using Radioimmunoprecipitation assay (RIPA) buffer containing a protease inhibitor cocktail. Protein concentrations were determined by a BCA protein assay kit (P0009; Beyotime, Shanghai, China). Equal amounts of protein were separated via denaturing Sodium dodecyl sulfate (SDS)-polyacrylamide gel electrophoresis, transferred electrophoretically onto polyvinylidene difluoride (PVDF) membranes and blocked, and then incubated overnight at 4°C with primary antibodies directed against HMGB3 (ab75782; Abcam), CD36 (18836-1-AP; Proteintech, Manchester, UK), E-cadherin (14472 S, CST), Snail (3879 S, cell signaling technology [CST]), vimentin (SK2482712C, Invitrogen), and β-actin. Corresponding horseradish peroxidase-conjugated secondary antibody was subsequently added for 2 hours at room temperature, and protein bands were visualized by exposure to film. The relative protein levels were calculated based on β-actin as the loading control.

### The Cancer Genome Atlas Data Acquisition

We downloaded The Cancer Genome Atlas (TCGA) RNA-sequencing level 3 data via the University of California, Santa Cruz (UCSC) Cancer Genomics Browser (http://genome-cancer.ucsc.edu) for HMGB3 mRNA and miR-200b expression in liver cancer. Gene expression RNA-sequencing data included 371 primary liver tumor samples and 50 liver normal tissue samples, the miRNA mature strand expression RNA-sequencing data included 372 primary liver tumor samples and 50 liver normal tissue samples. The 2 data sets showed the expression level as log2 (x+1)-transformed RNA-Seq by Expectation-Maximization (RSEM) normalized counts.

### Survival Analysis

The overall survival analysis of HMGB3 mRNA expression in liver cancer, from the TCGA data, was performed through the OncoLnc database in http://www.oncolnc.org. The bottom 20% of expression was defined as the low-expression group, and the top 80% comprised the high-expression group. The correlations of overall survival and disease-free survival analysis with miR-200b were conducted through Kaplan-Meier plotter for liver cancer, in the http://kmplot.com/analysis web site, using the microarray platform of Noncommercial spotted (GSE31384). As the system automatically selected the best cutoff, 0.85 of the miR-200b expression level was used to separate the high/low patient group.

### Statistical Analysis

All statistical analyses were performed using the SPSS version 19.0 statistical software package (IBM SPSS, Armonk, New York). Data are shown as the mean (standard deviation) of at least 3 independent experiments. Expression differences in samples and differences between different experimental groups were analyzed using the independent-samples *t* test. A *P* value of less than .05 was considered significant.

## Results

### Overexpression of HMGB3 and Low Expression of miR-200b in HCC Correlate With Poor Prognosis

**Figure 1.**
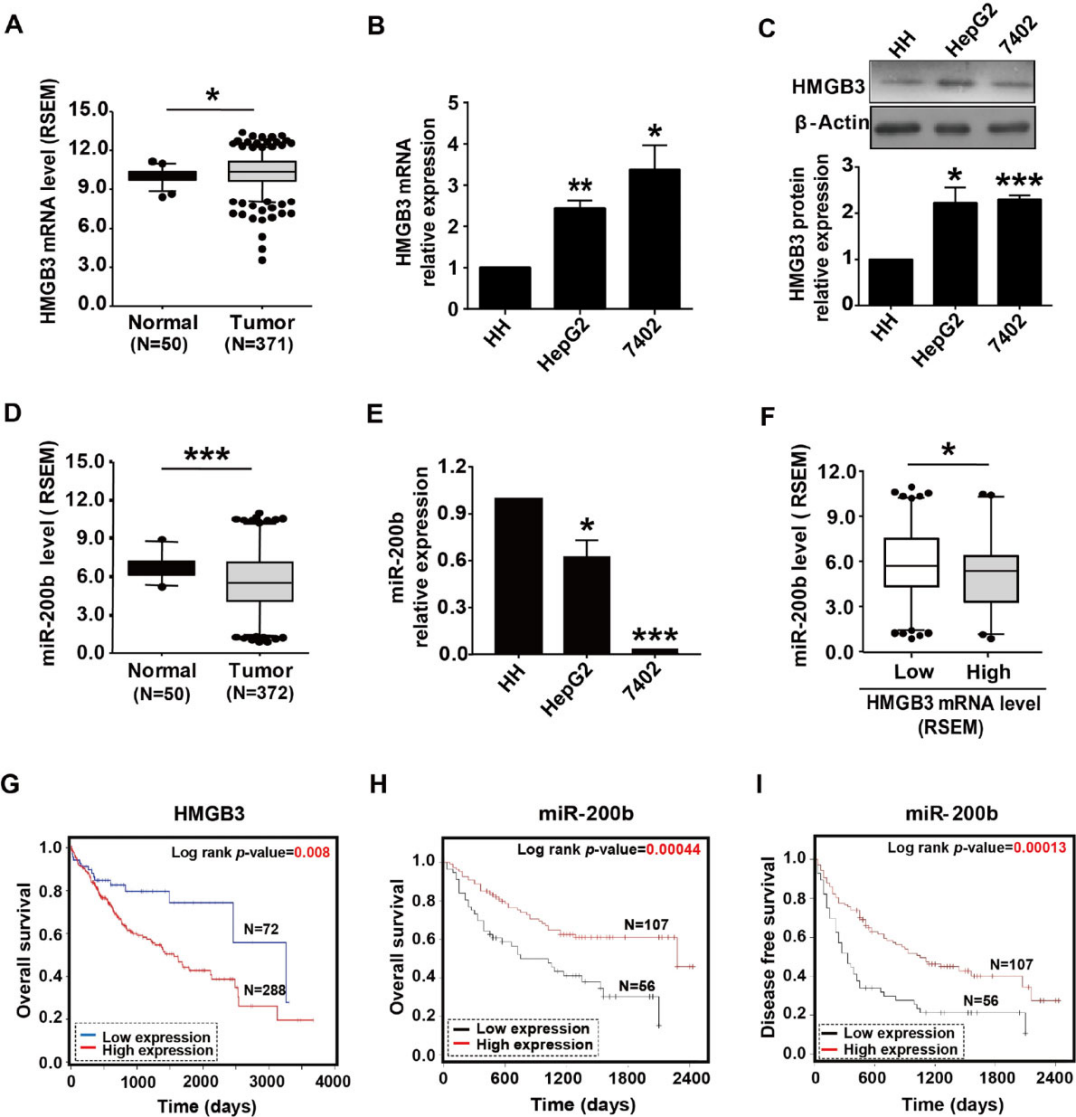
HMGB3 and miR-200b expression in HCC is associated with prognosis. A, HMGB3 mRNA levels in 50 normal liver tissues and 371 HCC tissues (*P* = .018). B and C, Relative expression levels of HMGB3 mRNA and protein in human liver cancer cell lines and in normal human liver cells (*P < .05; **P < .01). D, MiR-200b expression levels in 50 normal liver tissues and 372 HCC tissues (*P* = .000). E, Relative expression levels of miR-200b in human liver cancer cell lines and in normal liver cells (*P < .05; **P < .01). F, Correlation between HCC with high and low expression of HMGB3 and miR-200b expression (*P* = .014). G, Kaplan-Meier curves of overall survival time of patients with HCC based on HMGB3 expression in HCC samples obtained from the TCGA database. Three hundred sixty patients with HCC were recorded in the analyses. H and I, Kaplan-Meier survival analysis of the overall survival time and disease-free survival of patients with HCC based on miR-200b expression in HCC samples. One hundred sixty-three patients with HCC were recorded in the analyses. HCC indicates hepatocellular carcinoma; TCGA, The Cancer Genome Atlas database.

By analyzing normal liver tissue (n = 50) and HCC cases (n = 371) released by TCGA database, we found that HMGB3 expression was higher in the tumor group compared to the normal group (*P* = .018; Figure 1A). Next, we examined the expression levels of HMGB3 in the normal liver HH cell line, and HCC cell lines HepG_2_ and 7402, by both real-time qPCR and Western blot analysis. The results showed that HMGB3 expression levels were upregulated in HepG_2_ and 7402 cells when compared to normal liver HH cells (Figure 1B, C). In contrast, the miR-200b expression level was decreased in the cancer tissues (n = 372) compared to normal liver tissue (n = 50), based on the relevant tissue data from the TCGA database (*P* < .001; Figure 1D). We then performed qRT-PCR to evaluate miR-200b expression in HCC cells. Expression of miR 200b was lower in HepG_2_ cells and 7402 cells, as compared to normal liver HH cells (Figure 1E). These results are in agreement with preceding reports and demonstrate significant downregulation of miR-200b in HCC.^21,26^ We further analyzed whether there is a correlation between expression of HMGB3 and expression of miR-200b. The HMGB3 RSEM value of 10.825 from TCGA RNA-seq HCC tissues was used as the cutoff point to divide the HCC tissues into low (n = 241) and high (n = 126) HMGB3 expression groups. The level of miR-200b was significantly decreased with high HMGB3 expression (5.12 [2.27] vs 5.76 [2.36], *P* = .014) compared to the low-expression group of HMGB3, indicating that the expression of HMGB3 was negatively correlated with the level of miR-200b (Figure 1F).

Moreover, Kaplan-Meier curves showed that HMGB3 overexpression was significantly related to shorter overall survival (*P* = .008; Figure 1G). However, there were no significant correlations between HMGB3 overexpression and shorter disease-free survival (data not shown). We also found that miR-200b reduction was significantly associated with shorter overall survival (*P* = .00044; Figure 1H) and shorter disease-free survival (*P* = .00013; Figure 1I). Together, our data suggest that HMGB3 overexpression and miR-200b downregulation may play an important role in hepatocellular carcinogenesis.

### HMGB3 Is a Target of miR-200b in HCC Cell

**Figure 2.**
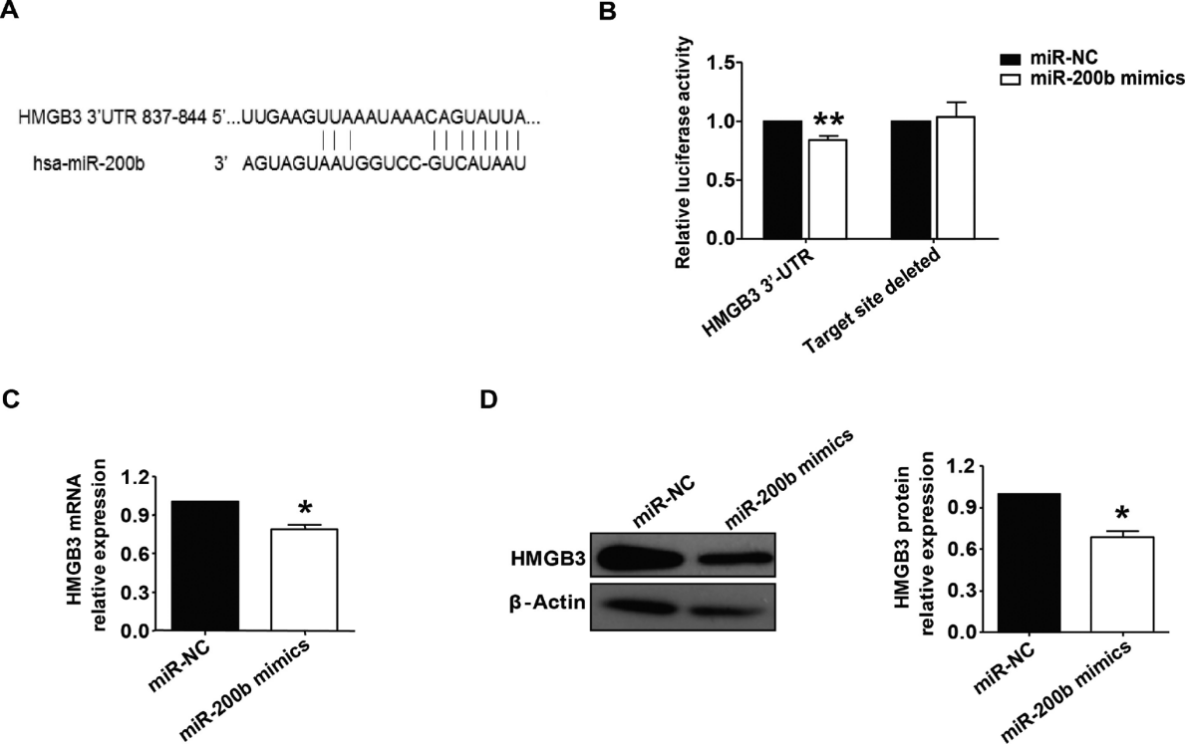
MiR-200b regulates HMGB3 expression by targeting the HMGB3 mRNA 3′-UTR in HCC. A, A schematic diagram of miR-200b and its target sites in the 3′-UTR of HMGB3. B, HepG_2_ cells were cotransfected with reporter plasmids with or without the miR-200b binding sequences in the 3′ UTR segment of HMGB3 mRNA. Luciferase reporter activity assay was performed 48 hours following cotransfection (**P < .01). C, Expression levels of HMGB3 mRNA, analyzed by qRT-PCR, in HepG2 cells after transfection with miR-200b mimics (*P < .05). D, Expression levels of HMGB3 protein in HepG_2_ cells, after transfection with miR-200b mimics, were analyzed by Western blotting (*P < .05). HCC indicates hepatocellular carcinoma; mRNA, messenger RNA; qRT-PCR, quantitative real-time polymerase chain reaction; UTR, untranslated region.

According to TargetScan analysis, we found that HMGB3 is a possible target for miR-200b. To confirm this, we constructed luciferase reporter plasmids containing either the binding sequences of miR-200b or the target site deleted sequences of miR-200b 3′ UTR target segments of HMGB3 mRNA, and cotransfected the plasmids into HepG_2_ cells along with miR-200b mimics or miR-NC. Dual luciferase reporter gene assays revealed that overexpression of miR-200b in HepG_2_ cells inhibited luciferase activity of the HMGB3 3′-UTR reporter gene, followed by a 16.4% downregulation (*P* < .01). However, no significant effect on luciferase activity of the target site deleted reporter genes was observed (Figure 2A, B). We then explored expression of HMGB3 in HepG_2_ cells with or without overexpression of miR-200b. By qRT-PCR and Western blotting, we found that both mRNA and protein expression of HMGB3 were reduced following transfection with the miR-200b mimics (Figure 2C, D). The above results indicate that miR-200b regulated HMGB3 expression in HepG_2_ cells.

### Silencing of HMGB3 Inhibits HepG_2_ Cell Proliferation and Migration

**Figure 3.**
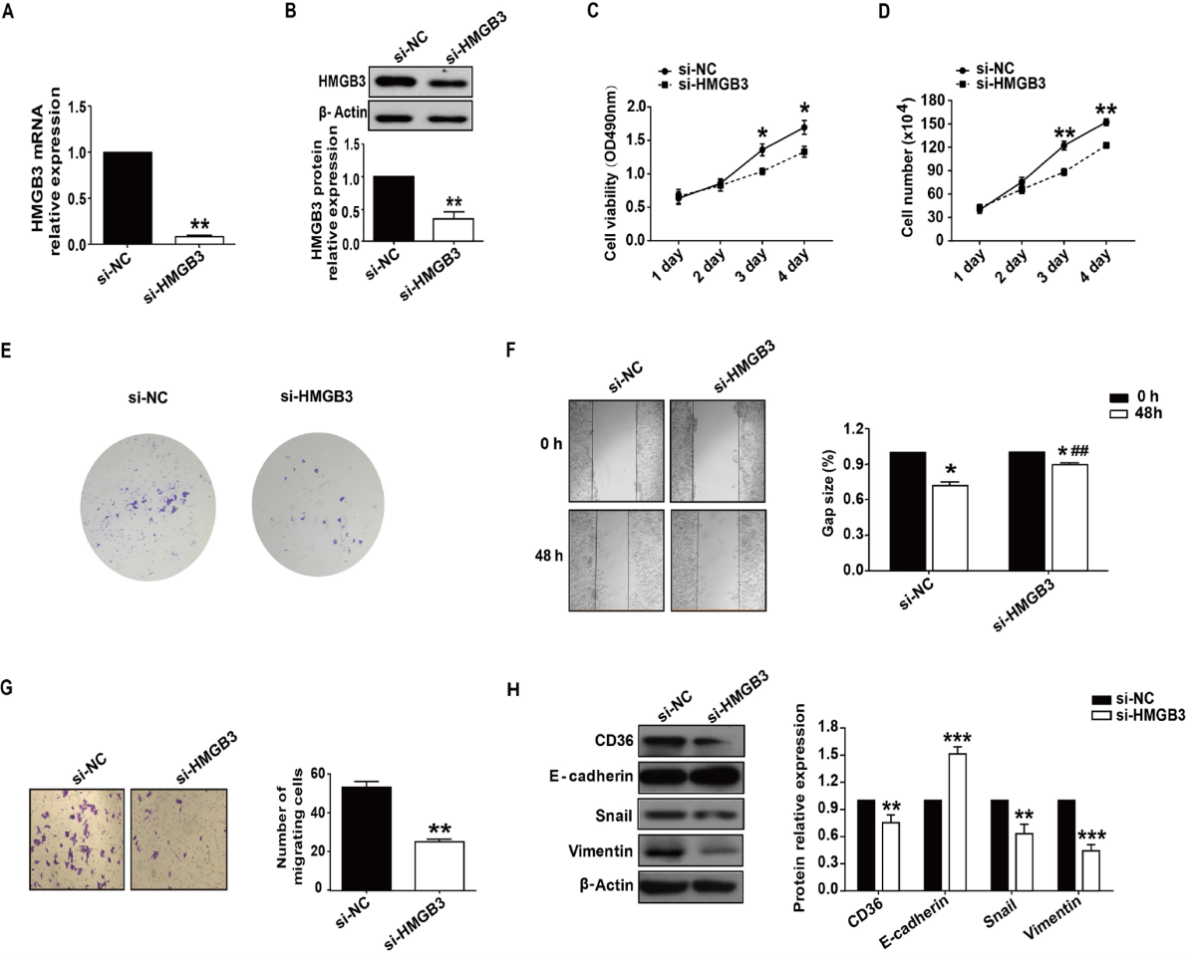
Silencing of HMGB3 inhibits HepG_2_ cell proliferation and migration. A and B, HMGB3 expression was demonstrated by qRT-PCR and Western blotting in HepG_2_ cells after transfection with an siRNA targeting HMGB3. C, MTT assays were used to determine the effect of RNA interference targeting HMGB3 on inhibition of tumor cell proliferation in HepG2 cells. D and E, Knockdown of HMGB3 inhibited growth and colony formation of HepG2 cells *in vitro*. F, HepG2 cells were wounded by a plastic tip after being transfected with siRNA-NC or siRNA-HMGB3. The wound was photographed at time 0 hour (upper) and 48 hours (lower). G, *In vitro* migration assay of HepG_2_ cells after transfection with siRNA-NC or siRNA-HMGB3 48 hours prior to cell inoculation into the upper chamber of a 24-well transwell. H, Compared to control siRNA, Western blot for CD36, E-cadherin, Snail and vimentin after transfection with siRNA-HMGB3. *P < .05; **P < .01; ***P < .001, as compared to the control siRNA group. qRT-PCR indicates quantitative real-time polymerase chain reaction; siRNA, small interfering RNA.

To examine the role of HMGB3 in hepatocellular carcinogenesis, siRNA targeting HMGB3 was used. RNA interference effectively reduced HMGB3 expression at both the mRNA and protein levels (Figure 3A, B). MTT assay showed that knockdown of HMGB3 significantly inhibited HepG_2_ cell growth at 72 and 96 hours after transfection (Figure 3C). This result is in accordance with the consequences of cell-growth curve and colony formation assay in HepG_2_ cell (Figure 3D, E). Subsequently, a scratch wound healing assay was used to evaluate the impact of HMGB3 on HCC cell migration. Confluent monolayers of HepG_2_ cells were scratched and cultured for 48 hours. As shown in Figure 3F, HepG_2_ cells transfected with NC siRNA spread into 28% of wound area, while cells downregulated for HMGB3 spread into 10.8% of the wound area. Consistent with this finding, a transwell assay also demonstrated knockdown of HMGB3 decreased migration of HepG2 cells (Figure 3G). We further analyzed the changes of the migration-related molecule, CD36. CD36 protein levels were downregulated in HepG2 cell lines following HMGB3 knockdown (Figure 3H). In addition, knockdown of HMGB3 protein resulted in the upregulation of E-cadherin and downregulation of both Snail and vimentin at the protein level (Figure 3H). The above results indicate that HMGB3 regulates cell proliferation and migration in HepG_2_ cell.

### Elevated Expression of miR-200b Inhibits HepG_2_ Cell Proliferation and Migration

**Figure 4.**
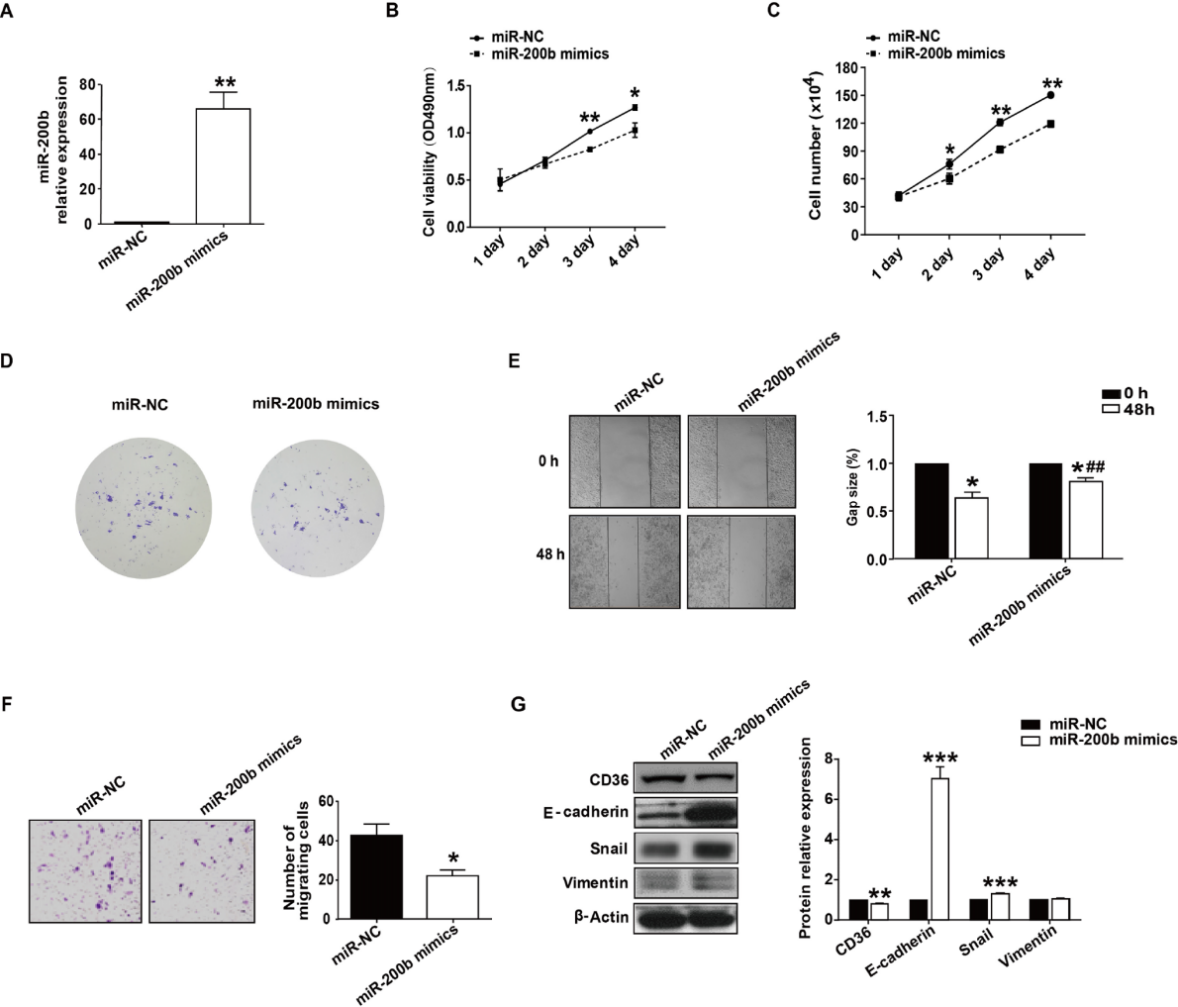
Upregulation of miR-200b inhibits HepG_2_ cell proliferation and migration. A, MiR-200b expression was shown by qRT-PCR in HepG_2_ cells following transfection with miR-200b mimics. B, MTT assays were used to examine the effect of miR-200b overexpression on inhibition of tumor cell proliferation in HepG2 cells. C and D, Transfection of miR-200b mimics repressed growth and colony formation of HepG2 cells *in vitro*. E, The HepG2 monolayer was scratched with a plastic tip after transfection with miR-NC or miR-200b mimics. The wound was photographed at time 0 hour (upper) and 48 hours (lower). F, *In vitro* migration assay of HepG2 cells after transfection with miRNA-NC and miR-200b mimics 48 hours prior to inoculation into the upper chamber of a 24-well transwell. G, Western blot analysis for CD36, E-cadherin, Snail and vimentin after transfection with miR-200b mimics. *P < .05; **P < .01; ***P < .001, as compared to the miR-NC group. miRNA indicates MicroRNA; qRT-PCR, quantitative real-time polymerase chain reaction.

Then, the effects of miR-200b on proliferation were investigated in HepG_2_ cells transfected with miR-200b mimics. Quantitative RT-PCR analysis confirmed that miR-200b expression was upregulated in HepG_2_ cells after transfection with miR-200b mimics when compared to miR-NC (Figure 4A). MTT assay results indicated that compared to miR-NC, cell proliferation was reduced at 72 and 96 hours in miR-200b-transfected HepG2 cells (Figure 4B). This finding is in line with the results of cell-growth curve and colony formation assay (Figure 4C, D). Wound healing assays showed that HepG2 cells transfected with miR-NC spread into 35.8% of wound area, while cells upregulated for miR-200b spread into 18.7% of the wound area (Figure 4E). Moreover, the Transwell assay also demonstrated miR-200b overexpression inhibited migration of HepG_2_ cells (Figure 4F). As CD36 may function in migration, we therefore inspected CD36 expression after transfection with miR-200b mimics. Our results showed miR-200b overexpression caused a significant reduction in CD36 protein level (Figure 4G). Moreover, overexpression of miR-200b brought about the upregulation of E-cadherin (Figure 4G). However, there was a slight upregulation in both Snail and vimentin at the protein level after transfection with miR-200b mimics. The above results indicate that miR-200b regulates cell proliferation and migration of HepG_2_ cells.

## Discussion

HMGB3, a member of the HMG-box family, is highly expressed in different types of cancers, including gastric cancer, esophageal squamous cell carcinoma, breast cancer, and urinary bladder cancer.^13-15,27^ Recent studies indicate that HMGB3 is associated with tumorigenesis. In leukemia, Petit *et al* discovered an oncogenic NPU98-HMGB3 fusion protein.^28^ HMGB3 also functions in the progression of colorectal cancer by regulating the Wnt/β-catenin pathway^16^ and overexpression is related with worse prognosis in gastric adenocarcinoma.^13^ In this study, we analyzed HMGB3 expression in HCC. Entries for HCC in the TCGA database show that, compared to normal tissue, the HMGB3 mRNA level in HCC is higher. Our qRT-PCR and Western blot results also show that HMGB3 expression levels are upregulated in HepG_2_ and 7402 cells as compared to normal liver cells. More importantly, we analyzed the expression of HMGB3 in tumors from 360 patients with HCC, in the TCGA database, and found that HMGB3 expression is negatively associated with overall survival time for patients with HCC. Compared to patients with low HMGB3 levels, the high-expressing HMGB3 patients display significantly lower survival time. HMGB3 knockdown impairs proliferation and migration, and significantly decreases expression levels of CD36, a transmembrane glycoprotein previously reported to promote cancer cell migration in human melanoma.^29^ Recently, epithelial–mesenchymal transition (EMT) has been demonstrated to play an important role in cancer metastasis.^30,31^ For instance, upregulation of Twist, a transcription factor that drives EMT, is related to HCC cell invasion and metastasis.^32^ In addition, E-cadherin loss in some cell types can also trigger an EMT and a large range of transcriptional and signaling changes that result in metastatic dissemination.^33^ Therefore, we detected EMT markers E-cadherin and Snail and vimentin and found HMGB3 knockdown led to the upregulation of E-cadherin and downregulation of Snail and vimentin at the protein level.

MiR-200b plays an inhibitory role in many tumors, including HCC, by targeting DNA methyltransferase 3a, RhoA and BMI1.^21,34,35^ By bioinformatic analyses and experimental verification, we identified HMGB3 as a target for miR-200b. We demonstrate an ability of miR-200b to downregulate HMGB3 expression by dual luciferase reporter gene assays and also show that both mRNA and protein expression of HMGB3 are reduced following miR-200b overexpression when the HMGB3 mRNA 3′UTR contains the miR-200b binding site, but not when the miR-200b binding site is deleted. Thus, the abovementioned results indicate that miR-200b regulates HMGB3 expression by targeting its 3′-UTR.

We further investigated the role of miR-200b in HCC. Here, we demonstrate that miR-200b is downregulated in HCC cells, a finding that is in agreement with previous reports.^21,26^ In addition, our data show that miR-200b downregulation is significantly associated with shorter overall survival and shorter disease-free survival. A critical role for the aberrant expression of miR-200b in hepatocarcinogenesis is supported by our finding that miR-200b overexpression in HepG_2_ cells results in suppression of cell growth and migration, and reduces CD36 expression levels, similar to knockdown of HMGB3. Moreover, miR-200b overexpression causes the upregulation of E-cadherin but only slight upregulation of Snail and vimentin at the protein level. Considering the relatively weak effect induced by miR-200b transfection on HMGB3 expression, we believe that other mechanisms are involved in the biological effects of miR-200b on HCC. An increasing number of studies have found various miR-200b targets, including ZEB2, PKCα, and Bmi-1, which are expressed in gastric adenocarcinoma, pituitary tumors, and prostate cancer, respectively.^23,36,37^ Therefore, we consider that other miR-200b-targeted proteins may play a role in the functional effects caused by miR-200b on HCC, and also lead to the upregulations of E-cadherin and Snail and vimentin.

In summary, our results show that HMGB3 overexpression is associated with patient with HCC survival, and miR-200b can inhibit tumor progression via directly targeting HMGB3 in HCC. These results suggest that HMGB3 may be a promising predictive biomarker and potential therapeutic target for HCC.

## Abbreviations

cDNA: complementary DNA
EMT: epithelial–mesenchymal transition
HCC: hepatocellular carcinoma
HMG-box: high-mobility group box; miRNA, micro RNA
mRNA: messenger RNA
NC: negative control
qRT-PCR: quantitative real-time polymerase chain reaction
PBS: phosphate-buffered saline
siRNA: small interfering RNA
TCGA: The Cancer Genome Atlas
UTR: untranslated region.
